# Antifungal Potential of *Diaporthe* sp. Endophytes from Antillean Avocado Against *Fusarium* spp.: From Organic Extracts to In Silico Chitin Synthase Inhibition

**DOI:** 10.3390/jof12010052

**Published:** 2026-01-11

**Authors:** Angie T. Robayo-Medina, Katheryn Michell Camargo-Jimenez, Felipe Victoria-Muñoz, Wilman Delgado-Avila, Luis Enrique Cuca, Mónica Ávila-Murillo

**Affiliations:** 1 Grupo de Investigación Estudio Químico de Productos Naturales Vegetales Bioactivos—QUIPRONAB, Departamento de Química, Facultad de Ciencias, Universidad Nacional de Colombia, Sede Bogotá, Bogotá D.C. 111321, Colombia; kmcamargoji@unal.edu.co (K.M.C.-J.); wadelgadoa@unal.edu.co (W.D.-A.); lecucas@unal.edu.co (L.E.C.); 2 Grupo de Investigación FarmaBioTech, Facultad de Ciencias Exactas y Naturales, Fundación Universitaria Salesiana, Bogotá D.C. 111071, Colombia; daniel.victoria@salesiana.edu.co

**Keywords:** *Persea americana* var. *americana*, *Diaporthe* sp., *Fusarium solani*, *Fusarium equiseti*, chitin synthase, molecular docking

## Abstract

Fungal endophytes have emerged as a promising source of bioactive compounds with potent antifungal properties for plant disease management. This study aimed to isolate and characterize fungal endophytes from Antillean avocado (*Persea americana* var. *americana*) trees in the Colombian Caribbean, capable of producing bio-fungicide metabolites against *Fusarium solani* and *Fusarium equiseti*. For this, dual culture assays, liquid-state fermentation of endophytic isolates, and metabolite extractions were conducted. From 88 isolates recovered from leaves and roots, those classified within the *Diaporthe* genus exhibited the most significant antifungal activity. Some of their organic extracts displayed median inhibitory concentrations (IC_50_) approaching 200 μg/mL. To investigate the mechanism of action, in silico studies targeting chitin synthase (CS) were performed, including homology models of the pathogens’ CS generated using Robetta, followed by molecular docking with Vina and interaction fingerprint similarity analysis of 15 antifungal metabolites produced by *Diaporthe* species using PROLIF. A consensus scoring strategy identified diaporxanthone A (**12**) and diaporxanthone B (**13**) as the most promising candidates, achieving scores up to 0.73 against *F. equiseti*, comparable to the control Nikkomycin Z (0.82). These results suggest that Antillean avocado endophytes produce bioactive metabolites that may inhibit fungal cell wall synthesis, offering a sustainable alternative for disease management.

## 1. Introduction

Species of the *Fusarium* genus rank among the most detrimental phytopathogens globally, causing toxin contamination as well as severe diseases and economic losses across a broad spectrum of crops, including cereals, vegetables, and tropical fruits [1,2]. These pathogens induce chlorosis and cotyledon necrosis, water-soaked lesions on the crown and lower stem, pre-emergence and post-emergence growth retardation, brown to black wilt in the lower main root and lateral roots, vascular damage, and, in some cases, produce mycotoxins harmful to human and animal health [3,4].

Current strategies for controlling *Fusarium* infections encompass the use of resistant crop varieties, implementation of best agricultural practices, and application of synthetic fungicides like phosphine, formaldehyde, carbendazim, thiophanate-methyl, propiconazole + prochloraz, among others [5,6]. However, chemical interventions raise critical concerns regarding environmental sustainability, potential toxicity, and the accumulation of harmful residues in food products [7]. In response to these challenges, plant–microbiome interactions, particularly those involving fungal endophytes, have emerged as a promising avenue for sustainable disease management, due to their ability to enhance plant resilience against both biotic and abiotic stressors [8,9,10].

Notably, the selection of host plants plays a key role in the successful isolation of endophytes with the highest bioactive potential, often found in plants from unique ecosystems like tropical forests, endemic species with unusual longevity, or those that have adapted to extreme environmental conditions [11,12]. That is the case of Antillean avocado (*Persea americana* var. *americana*) growth in the Montes de María region (Colombian Caribbean). The endemic ecotypes (Cebo, Leche, and Manteca)—distinguished based on fruit characteristics, including pulp texture and fat content—display three key characteristics for endophytic diversity: (1) propagation by seed, (2) development in tropical regions, and (3) survival under adverse sanitary conditions. Antillean avocado plants have been propagated by seed over five decades, leading to their classification as endemic varieties well adapted to the region’s distinct climatic and geographic conditions (0–1000 m.a.s.l. and 18–27 °C) [13,14]. Remarkably, the phenomenon of vertical transmission of endophytes, through seeds, ensures their presence in subsequent plant generations and plays a crucial role in host survival. This mutualism provides additional protection against pathogen attacks, enhancing plant growth and development [15,16]. In addition, tropical and subtropical regions host most of the world’s plant biodiversity, and as a result, the diversity of endophytic microorganisms in these climatic zones is also remarkably high. Plants grown from seeds in natural tropical environments are exposed to a wider array of microbial sources and local biotic interactions, which can foster a richer and potentially more specialized endophytic community with capacity for producing diverse bioactive compounds [17].

Unfortunately, the cultivation of these endemic ecotypes has been severely affected by the Avocado Wilt Complex (AWC), a group of phytopathogens including *Fusarium*, *Verticillium*, and *Phytophthora*, which aggressively compromise the root systems of most avocado trees [18,19]. It is well established that the presence of endophytes shapes the phenotype of host plants through mutualistic plant–microbiome interactions. Plants free of microbes would hardly survive under natural conditions [20]. Endophytes offer their host numerous advantages against biotic stress by producing antimicrobial secondary metabolites and enhancing the systemic response to pathogen presence [21]. For example, endophytic fungi such as *Trichoderma* spp. have been widely reported to suppress soil-borne pathogens through mycoparasitism, competition, and the production of antifungal metabolites, contributing to disease control in maize, cabbage, cotton, wheat, and other important crops [22,23]. Similarly, endophytic *Chaetomium* species have demonstrated strong antagonistic activity against important plant pathogens, such as *Fusarium* spp. and *Phytophthora* spp., by secreting antifungal secondary metabolites and cell wall-degrading enzymes [24]. In consequence, the survival of some avocado plants suggests the presence of intrinsic defense mechanisms, potentially mediated by a diverse and functionally significant endophytic community [25,26].

In the same way, understanding the mechanisms of action of antifungal compounds is fundamental for advancing plant disease management and addressing the growing challenge of fungal resistance. Antifungal compounds target essential structures and metabolic processes in phytopathogenic fungi through multiple mechanisms of action like intracellular reactive oxygen species accumulation, cell membrane disruption, lipid peroxidation, mitochondrial dysfunction [27,28], inhibition of cell wall biosynthesis [29,30], etc. In particular, in the context of plant pathogenic fungi like *Fusarium* spp., targeting chitin synthase (CS) offers several specific advantages compared to other antifungal targets [31,32]. The unique composition of the fungal cell wall—dominated by polysaccharides such as glucans and chitins, along with glycoproteins—distinguishes it fundamentally from human cellular structures [33]. In addition, chitin is an indispensable structural polysaccharide in the fungal cell wall, providing mechanical strength and integrity that are critical for hyphal growth, morphogenesis, and pathogenicity [34,35]. Consequently, CS, the enzyme responsible for chitin polymerization, has emerged as a strategic molecular target for the development of selective antifungal agents [36,37,38]. Previous studies focused on CS inhibitors from fungal endophytes have demonstrated their capacity as antagonists of this target [39,40,41,42]. For example, extracts from fungal endophytes isolated from *Protium heptaphyllum* and *Trattinnickia rhoifolia* demonstrated significant antagonism against *Fusarium oxysporum*, implicating the secretion of antifungal compounds with potential effects on cell wall biosynthesis enzymes [39]. Similarly, a combined in vitro and in silico investigation of the endophyte *Bacillus velezensis* CBMB205 revealed novel antifungal activity against *F. oxysporum* f.sp. *cubense*, where metabolite–target interactions predicted the inhibition of CS and 1,3-glucan synthase, two essential fungal enzymes involved in cell wall synthesis [40]. Studies on *Talaromyces oaxaquensis* further show that the secretion of antifungal metabolites contributes to antagonistic activity, consistent with mechanisms that disrupt structural integrity in *F. oxysporum* f.sp. *cubense*. Furthermore, the discovery of Chaetoatrosin A, a novel CS II inhibitor produced by *Chaetomium atrobrunneum* F449, provides direct evidence that endophytic fungi can synthesize compounds capable of inhibiting CS activity [42]. Nevertheless, despite its clinical and agricultural relevance, structural data for CS in specific phytopathogens such as *Fusarium solani* and *Fusarium equiseti* remain limited, often hindering the rational elucidation of inhibition mechanisms.

In this context, the present study aims to investigate the microbiota associated with Antillean avocado trees in Montes de María—Colombian Caribbean—and to evaluate their potential as antifungal metabolite producers, with inhibitory activity against *F. solani* and *F. equiseti.* In addition, we hypothesize the molecular basis of the antifungal activity observed in this study, through an in silico approach. Based on the results from the in vitro assays, we performed homology modeling to generate 3D structures of the CS enzymes for both pathogens, followed by molecular docking and interaction fingerprint (PLIF) analyses. This computational strategy was employed to evaluate whether bioactive metabolites, previously isolated from endophytic *Diaporthe* species, could act as potential inhibitors of this critical enzymatic target.

## 2. Materials and Methods

### 2.1. Collection of Plant Material

Leaves and secondary roots from the Cebo, Manteca, and Leche ecotypes of avocado (*Persea americana* var. *americana*) cultivated in the Caribbean region of Colombia (09°34′42″ N 75°16′15″ W) were collected in 2017. The collection of plant material and the study of the isolated fungi were covered by Permission No. 121 of 22 January 2016 (Amendment No. 21), under the Access to Genetic Resources and Derivative Products Agreement No. RGE 46 (Article 6—Law 1955 of 2019), granted by Ministerio de Ambiente y Desarrollo Sostenible de Colombia. Fungal isolates were placed in the QUIPRONAB strain collection, located at the Chemistry Department of Universidad Nacional de Colombia, Sede Bogotá.

Leaves and secondary roots were indeed collected equitably from each ecotype (Cebo, Manteca, and Leche). Additionally, leaves were collected from three different canopy levels to account for microenvironmental variations in light intensity and relative humidity [43], and were then pooled into a single sample per ecotype. Endophytic fungi were isolated from plant material obtained from healthy, long-lived trees that had survived under adverse sanitary conditions in this region, ensuring that the selected individuals were representative of naturally resilient hosts.

### 2.2. Avocado Phytopathogen Isolation

*Fusarium* pathogens were isolated from secondary roots of trees exhibiting symptoms of AWC. Plant material was surface sterilized following established protocols [44], and fungal isolates were identified through a combination of morphological and molecular characterization [45].

Two *Fusarium* isolates were selected based on an in vivo pathogenicity test. Avocado seedlings were germinated from Antillean avocado seeds and cultivated under controlled glasshouse conditions (~18–20 °C day, ~12–15 °C night) for four months, which then reached 40–50 cm and developed at least four true leaves [46]. Each treatment included ten plants. Conidial suspensions (1 × 10^7^ CFU/mL) were prepared from 14-day-old cultures grown on potato dextrose agar (PDA), and roots were immersed for 2 min prior to transplanting into sterile soil. An additional 1 mL of inoculum was applied near to the root zone. Sterile distilled water served as a negative control. Plants were irrigated weekly for six weeks, and disease severity was evaluated using a 0–3 scale: 0 (no lesion), 1 (chlorosis/slight withering), 2 (severe withering/defoliation), and 3 (plant mortality). The disease index (DI) was calculated using DI = 100 × [(a.X0) + (b.X1) + (c.X2) + (d.X3)]/(N × 3), where a–d represents the number of plants at each infection level, and N is the total plants per treatment. Following the experiment, seedlings were uprooted, and the causal agents were re-isolated via root surface sterilization and subsequent culture on PDA.

### 2.3. Fungal Endophytes Isolation

Healthy leaves and secondary roots were collected from three Antillean avocado ecotypes—Cebo, Leche, and Manteca—that had survived adverse phytosanitary conditions in the Colombian Caribbean region. Plant material was surface sterilized following the previously described protocol. The samples were excised into 5 mm^2^ segments [47] and plated onto different solid culture media, including V8 juice agar, malt extract agar, yeast glucose extract agar, and potato dextrose agar, all supplemented with chloramphenicol (400 ppm). Following the incubation period, individual fungal morphotypes were subcultured onto PDA to obtain pure isolates. To establish a well-characterized fungal collection and ensure culture purity, axenic cultures were obtained [48]. Endophytic fungal strains were subsequently preserved under cryogenic storage conditions [49] for further study.

### 2.4. Inhibitory Activity of Fungal Endophytes and Their Organic Extracts

Dual culture assays were conducted on PDA plates (ø = 90 mm) by placing 5 mm agar plugs of the entophytic isolate and the phytopathogen (*F. solani* or *F. equiseti*) 50 mm apart. Cultures were incubated at 28°c for 14 days and radial fungal growth was measured daily. Control plates consisting of the pathogen grown under identical conditions without endophyte interaction were included for comparison [50]. The experiment was performed in triplicate, and only endophytes exhibiting significant inhibitory effects on pathogen growth were selected for subsequent organic extraction.

Selected endophytic isolates were cultured in 250 mL Erlenmeyer flasks containing 100 mL of 2% yeast extract broth and incubated at 28 °C with constant agitation (150 rpm) for 14 days. Biotechnological products were extracted via three consecutive ultrasound-assisted extractions using 300 mL of ethyl acetate (EtOAc) per flask. The organic phase was subsequently filtered, separated, and concentrated under reduced pressure to obtain crude extracts [51].

Fungal extracts were tested for antifungal activity using agar dilution method in 12-well plates. A stock solution (50 mg/mL) was prepared in ethanol, from which 40 µL was combined with 10 µL of MTT (2 mg/mL)—to facilitate visualization of the colony edge within the wells—and 1950 µL of PDA, resulting in a final concentration of 1 mg/mL per well. Pathogens were inoculated using a sterile toothpick from a seven-day PDA culture and incubated at 28 °C for 72 h. Mycelial growth inhibition (% MGI) was quantified using ImageJ™ (Version 1.53u) and compared to untreated controls [52]. Extracts exhibiting (% MGI) ≥ 50% at 1000 µg/mL were selected for further dose–response analysis to determine IC_50_ values, employing the agar dilution method, with concentrations between 25 and 3000 µg/mL. This threshold was selected with basis on preliminary bioactivity screening studies of crude extracts [53,54] and widely adopted benchmarks for natural products’ antimicrobial activity established by Morales et al. [55]. All experiments were conducted in triplicate, and IC_50_ values were calculated using GraphPad Prism 7.05 software.

### 2.5. Morphological and Molecular Characterizations of Endophytes

Fungal endophytes exhibiting ≥50% MGI against *Fusarium* spp. were identified through a combination of morphological and molecular analyses. Molecular identification was conducted via DNA sequence analysis of the internal transcribed spacer (ITS-1) region of ribosomal DNA, following established protocol comparisons for the internal transcribed spacer ITS-1 of the ribosomal DNA [56]. The resulting sequences were queried against GenBank using the Basic Local Alignment Search Tool (BLAST) hosted at NCBI (https://blast.ncbi.nlm.nih.gov/Blast.cgi; URL accessed on 1 August 2023) to identify the closest taxonomic matches [57]. All ITS-rDNA sequences were submitted to GenBank to obtain accession numbers (Table S1).

### 2.6. Homology Modeling and Structural Selection

The amino acid sequences of CS for *F. solani* and *F. equiseti* were retrieved from the UniProtKB database [58]. To identify a suitable structural template, sequence alignment was performed using BLAST against the Protein Data Bank (PDB) to find a crystallographic structure with high identity and query coverage. Three independent automated prediction servers were employed to generate the 3D structural models: Robetta [59], AlphaFold [60], and I-TASSER [61]. To select the most reliable structure among the generated predictions, the models were structurally superimposed onto the identified template. The final selection was determined by calculating the Root Mean Square Deviation (RMSD) of the backbone atoms relative to the template; the model exhibiting the lowest RMSD value was chosen.

### 2.7. Molecular Docking and Interaction Analysis

The molecular docking simulations were performed using AutoDock Vina (v4.2.6) to predict the binding affinity and orientation of the isolated metabolites within the catalytic site of CS [62]. The selected 3D models of *F. solani* and *F. equiseti* CS were prepared by adjusting the protonation states at pH 7.4 and were assigned using PDB2PQR; partial charges were added [63]. The 3D structures of the 15 fungal metabolites were generated and energy-minimized using RDKit (MMFF94 force field) [64]. Gasteiger partial charges were assigned, and rotatable bonds were defined before conversion to PDBQT format using OpenBabel of all structures [65].

The search space was defined centered on the coordinates of the reference inhibitor Nikkomycin Z (derived from the template PDB: 7WJO) and creating a cubic grid box of 8Å in each dimension to cover the active site pocket [66]. Docking runs were executed with an exhaustiveness parameter of 16. To validate the protocol, a re-docking assay was performed using the co-crystallized ligand, calculating the Root Mean Square Deviation (RMSD) between the predicted and experimental poses using spyrmsd [67]. The resulting protein–ligand complexes were analyzed to identify key molecular contacts. Interaction fingerprints (IFPs) were generated using ProLIF to map hydrogen bonds, hydrophobic contacts, and pi-stacking interactions [68]. To rank the compounds, a similarity analysis was conducted by calculating the Cosine coefficient between the interaction fingerprint of each metabolite and that of the reference inhibitor (Nikkomycin Z).

To identify the most promising candidates, a consensus scoring function (consensus score) was established by integrating the binding affinity and the interaction similarity. Since the Vina score (*nVS*) and the PROLIF similarity (*nCS_PROLIF_*) have different units and scales, both variables were normalized using the Min-Max scaling method. The consensus score for each molecule was calculated as the arithmetic mean of these normalized values, as shown in Equation (1).

(1) $$Consesus\;score=\frac{nVS+{nCS}_{PROLIF}}{2}$$

## 3. Results and Discussion

### 3.1. Avocado Phytopathogen Isolation

Six fungal strains were isolated from the secondary roots of Antillean avocado trees exhibiting symptoms of root rot. These isolates were subjected to morphological and molecular characterization. DNA sequencing was performed to facilitate taxonomic identification (Table 1).

**Figure 1:**
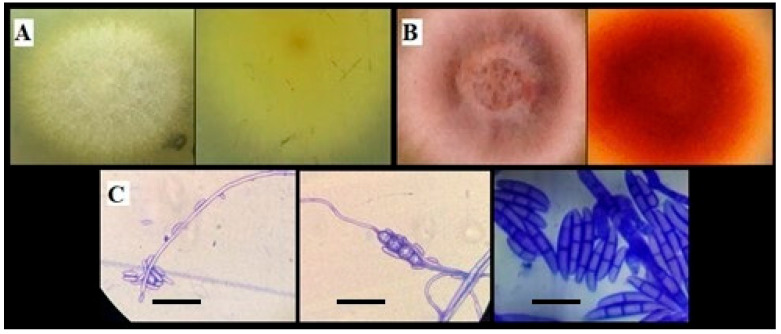
Morphological characterization of *Fusarium* pathogens isolated. (**A**) *Fusarium solani* grown on PDA after 7 days of incubation (obverse and reverse). (**B**) Colony (obverse and reverse) of *Fusarium equiseti* grown on PDA after 7 days of incubation. (**C**) Reproductive structures of *Fusarium* spp.; from left to right: microconidia (scale bar = 50 µm), chlamydospores (scale bar = 30 µm), and macroconidia (scale bar = 30 µm).

After pathogenicity assay on avocado seedlings, two *Fusarium* strains could induce disease symptoms. *F. solani* and *F. equiseti* exhibited a 100% disease incidence, with disease severity indices of 77% and 80%, respectively, six weeks post-inoculation. Koch’s postulates were confirmed through the successful re-isolation of the pathogens following surface disinfection of infected seedling roots and subsequent culture on PDA. Morphological identification was confirmed based on macroscopic and microscopic characteristics of the re-isolated strains. *Fusarium* spp. isolates were described as light yellow (UN29) and salmon-colored (UN37) colonies, whose pigments diffused into the culture medium, with velvety–cottony aerial mycelium and an approximate radial growth rate of 2–3 mm/day for both isolates. Microscopic characterization revealed colonies with abundant production of three different types of asexual spores, microconidia, chlamydospores, and macroconidia, which are considered the most effective means of reproduction and dispersal, as well as the primary source of infection in plants [69]. Microconidia were oval in shape and occurred in false heads; chlamydospores were observed as cells with a thin wall emerging from the hyphae; and macroconidia were identified as fusiform, multiseptated structures [70] (Figure 1).

These findings are consistent with previous reports on the phytopathogenic potential of *Fusarium* species [2,69,71]. Indeed, *Fusarium* spp. are among the most prevalent pathogens affecting avocado crops globally, with *F. solani*, *F. equiseti*, and *F. oxysporum* recognized as the most economically significant [69]. Typical disease symptoms include chlorosis, necrosis, water-soaked lesions, growth retardation, and root wilt [69]. In addition, previous studies have reported the disease incidence of *Fusarium* species in avocado seedlings [72,73,74]. For instance, a study conducted in Michoacán, Mexico, on *Persea americana* var. *drymifolia* identified *F. oxysporum* and *F. solani* isolates as pathogenic, with 63% causing wilting and 16% inducing necrosis [72]. Similarly, in Kenya, *F. solani*, *F. oxysporum*, and *F. equiseti* were associated with stem-end rot in Hass avocados, with disease severity ranging from 19.2% to 60.8% [73]. In South America, a study on the Hass variety with plants showing symptoms, such as leaf, branch, and stem necrosis, identified *F. solani* as the pathogen responsible for dieback [74]. Considering the aforementioned, our findings further establish *Fusarium* as a major pathogen in avocado cultivation.

Previous research has reported the presence of oomycetes like *P. cinnamomi*, *P. citricola*, and *P. heveae*, as well as fungal pathogens including *Lasiodiplodia theobromae*, *Pythium* spp., *Fusarium* spp., and *Verticillium* spp., in Hass avocado plantations in Antioquia, with disease incidence ranging from 10% to 65% [75]. Additionally, *Phytophthora heveae*, *Calonectria* spp., and *Fusarium* spp. have been identified in avocado nurseries in Valle del Cauca [76]. In *Persea americana* var. *americana* (Antillean avocado), *P. cinnamomi* has been identified as the primary causal agent of wilt, with an incidence exceeding 40% in 2009 [19]. Moreover, recent studies suggest that *Colletotrichum* spp. contribute to abnormal seedling development and formation of necrotic acervuli in nursery settings, along with other pathogenic organisms including *P. cinnamomi*, *Sclerotium rolfsii*, and *Nectria* spp. that have been implicated in symptoms such as wilting, necrosis, and terminal stem/root rot [77]. Although *Phytophthora cinnamomi* is a primary causal agent of root rot in Colombian avocado orchards [78,79], it was not recovered in this study probably due to its stringent nutritional and environmental requirements for in vitro growth [80,81], which can make culturing difficult. Therefore, its presence or absence in the sampled tissues should ideally be confirmed through molecular detection methods, such as PCR-based assays targeting species-specific markers [82].

### 3.2. Avocado Fungal Endophytes Isolation

Following surface disinfection and incubation of plant material, a total of 88 strains were selected as different morphotypes based on their growth on PDA. Of these, 19 endophytic fungi were isolated from avocado roots and 69 were recovered from leaves. Most isolates were obtained from Cebo and Manteca avocado ecotypes, whereas the Leche ecotype exhibited the lowest percentage of fungal endophytes (17%). This reduced endophyte diversity in Leche ecotype may be related to observations reported by local avocado growers, who indicate that this ecotype has been the most susceptible to phytopathogenic infections. Consequently, the Leche ecotype has become increasingly rare in the Montes de María region (Oral communication, Asociación de Productores de Aguacate Tecnificado de los Montes de María, Asproatemom, 2017). At this point, seed-based propagation of Antillean avocados could be determinant for the unexpected resistance of Cebo and Manteca ecotypes. Vertical transmission of endophytic microbes via seeds is increasingly recognized as an important mechanism by which beneficial symbionts are passed from one plant generation to the next [15]. Seed-borne endophytes are transmitted internally through the vascular tissues or reproductive structures of the parent plant, allowing them to colonize the embryo and persist in seedlings after germination [83,84]. Such vertically transmitted endophytes can contribute to plant fitness by producing phytohormones, enzymes, antimicrobial compounds, and other secondary metabolites that support growth and stress resilience under biotic and abiotic challenges [84,85]. Although the specific dynamics of vertical transmission in *Persea americana* remain to be fully elucidated, evidence across diverse plant species demonstrates that seed endophytes frequently represent a core portion of the seed microbiome that is inherited by offspring, providing an early microbial inoculum that may help seedlings enhance stress tolerance by producing antimicrobial metabolites and activating systemic defense mechanisms against pathogens [21].

**Figure 2:**
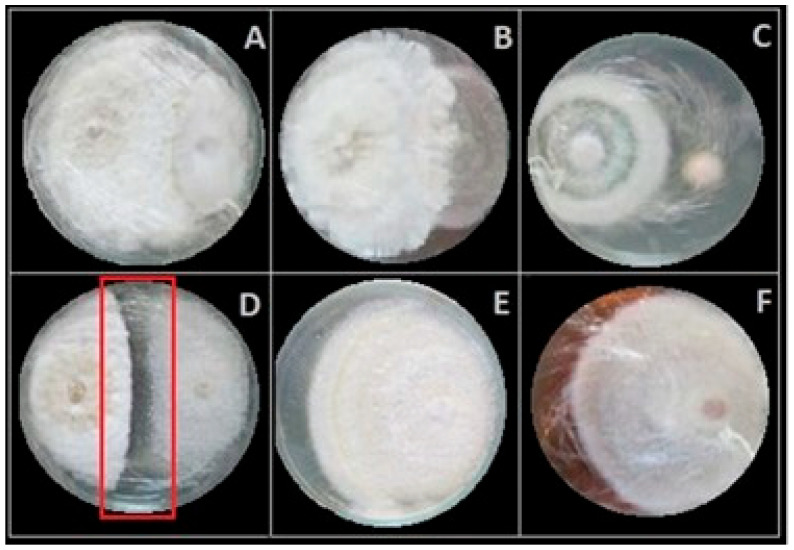
Biocontrol effects of fungal endophytes isolated from Antillean avocado leaves and roots, against *Fusarium* phytopathogens after 14 days of incubation (**left**: endophyte; **right**: pathogen). Antagonistic interactions observed: competition for space and nutrients (**A**), Mycoparasitism (**B**), Fumigant activity via volatile organic compounds (**C**), and antibiosis observed by mycelial growth inhibition into the red box (**D**). Growth control of *F. solani* (**E**) and *F. equiseti* (**F**).

The inhibitory activity of 88 fungal endophytes was assessed using dual culture assays. After 14 days of incubation, some avocado-associated endophytes exhibited four distinct antagonistic interactions: (1) Competition for space and nutrients, promoting high colonization density (Figure 2A). (2) Mycoparasitism, characterized by endophytic overgrowth on the pathogen’s mycelium and the formation of appressoria on hyphae (Figure 2B). (3) Fumigant activity via volatile organic compounds (VOCs), validated through separate inoculation and confrontation assays (Figure 2C) [86]. (4) Antibiosis, wherein extracellular enzymes, volatiles, and antibiotics inhibited pathogen growth without physical contact, showing clear zones between two fungi (Figure 2D).

Previous studies have highlighted the importance of chitin synthases for proper hyphal and pathogenesis of *Fusarium* spp. [87]. Therefore, in this study, antibiosis was used as the primary criterion to select isolates with potential CS inhibitory activity, resulting in 35 strains that inhibited the growth of *F. solani* and 29 strains active against *F. equiseti*. In addition, the production of secondary metabolites is generally enhanced during the late exponential or stationary growth phases, making it necessary to adjust harvest timing to optimize metabolite yield. Accordingly, most filamentous fungi are commonly cultured for approximately seven days prior to metabolite extraction to ensure these optimal physiological conditions are reached [51]. Given this, the 35 endophytes isolated from Antillean avocado leaves and roots that exhibited growth inhibition zones of *Fusarium* spp. in dual culture assays were incubated for 14 days prior to organic extraction using EtOAc.

**Figure 3:**
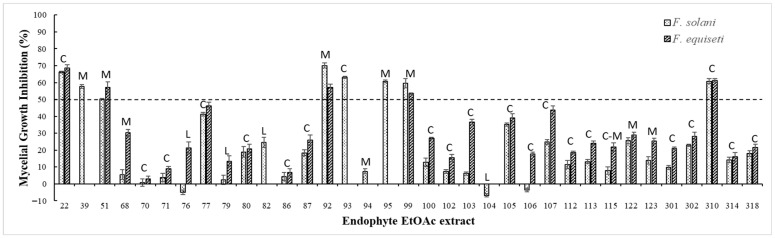
Origin of Antillean avocado endophytes that showed inhibitory activity on *F. solani* and *F. equiseti* growth in dual cultures. The graphic highlights those which their EtOAc extracts exhibited % MGI of at least one pathogen over 50% in the agar dilution test. C: *Cebo* ecotype; M: *Manteca* ecotype; L: *Leche* ecotype.

In an agar dilution assay, eight EtOAc extracts demonstrated >50% inhibition of at least one *Fusarium* pathogen, at 1000 µg/mL. However, extracts UN76, UN104, and UN106, derived from Leche and Cebo ecotypes, unexpectedly promoted *F. solani* growth (Table 2 and Figure 3). A plausible explanation for the enhanced growth of *Fusarium* pathogens observed with these endophytic extracts is the hormesis phenomenon, a well-documented biphasic dose–response in which low concentrations of a stressor stimulate biological activity while higher concentrations inhibit it [88]. Hormetic responses have been reported in a variety of fungal plant pathogens, where sub-inhibitory doses of fungicides or other bioactive compounds can paradoxically enhance mycelial growth and virulence before inhibitory effects occur at higher doses [89,90]. Although most hormesis research has focused on chemical fungicides, the underlying principle applies broadly across stressors, including natural metabolites, and has been highlighted as a potential factor complicating the use of antifungal agents in agriculture [91]. For example, low-dose exposure to fungicides such as carbendazim has been shown to stimulate mycelial growth and aggressiveness in *Magnaporthe oryzae*, demonstrating that biphasic responses to bioactive compounds can increase pathogen performance at sublethal concentrations [92].

The antifungal activity of the extracts evaluated in this study was comparable to that of other extracts described in the literature as promising inhibitors of *Fusarium* species. For example, the crude extract of the endophyte *Fusarium proliferatum*, obtained with EtOAc, showed 75.51% inhibition of *F. oxysporum* at a concentration of 1000 μg/mL [53]. Similarly, the EtOAc extract of the endophyte *Chaetomium globosum* exhibited mycelial growth inhibition percentages of 59.06%, 64.90%, and 59.41% against *F. graminearum*, *F. oxysporum*, and *F. moniliforme*, respectively [93].

The inhibitory concentrations (IC_50_) of the most promising extracts, those with MGI higher than 50% at 1000 µg/mL, were determined using a standard dose–response model, yielding values ranging from 199 to 688 µg/mL. Notably, the UN310 extract exhibited the highest antifungal activity, with an IC_50_ of 362 µg/mL against *F. equiseti* and 204 µg/mL against *F. solani*. These values were not significantly different from those of the UN93 extract, which displayed an IC_50_ of 200 µg/mL against *F. solani*. In contrast, the endophytic fungus UN51 demonstrated the weakest antifungal activity, with an IC_50_ exceeding 3000 µg/mL against both *Fusarium* pathogens (Table 3).

### 3.3. Morphological and Molecular Characterizations of the Most Promising Endophytes

**Figure 4:**
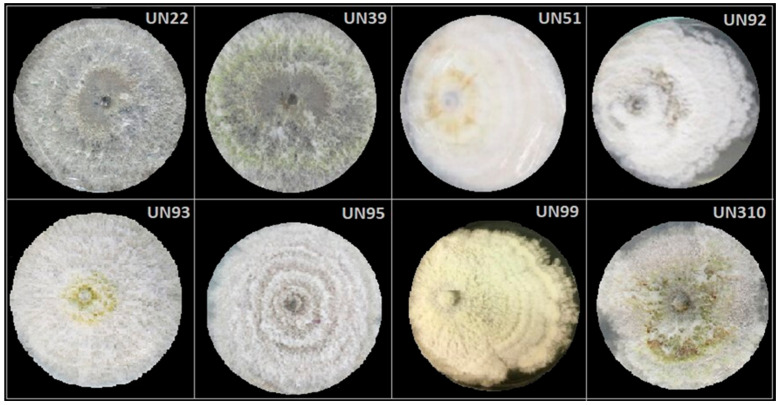
Macroscopic features of selected antifungal endophytes. Pictures were taken on PDA plates at 14th day of culture.

Figure 4 illustrates the macroscopic characteristics of the eight selected endophytic strains cultured on PDA. Colonies of UN22, UN39, UN92, UN93, UN95, UN99, and UN310 exhibited white to gray, wooly aerial mycelium, accompanied by yellow-orange exudates and a dark gray to black reverse pigmentation, with distinct lobed margins. In contrast, strain UN51 displayed the most divergent macroscopic features, forming a colony with entire margins, a light-yellow velvety mycelium, and occasional dark brown exudates.

A significant challenge in endophytic fungal identification is the absence of conidia or other reproductive structures when cultured in conventional media, as is widely reported in the scientific literature [43]. To overcome this limitation, molecular characterization was performed on the eight selected endophytes. Sequence analysis revealed a 99–100% similarity with previously deposited sequences in GenBank, except for strain UN51, which exhibited 96% correspondence (Table 4). All strains coded as UN22, UN39, UN92, UN93, UN95, UN99, and UN310 were closely related to species of the genus *Diaporthe*. In contrast, strain UN51 was more distant and showed greater similarity to the ITS1 region of species within the genus *Nodulisporium* (Figure 4). Given its IC_50_ value (Table 3), UN51 was reclassified as an inactive endophytic strain against *F. solani* and *F. equiseti.* Species of *Nodulisporium* are known to produce various secondary metabolites with herbicidal [94], insecticidal [95], cytotoxic, antiplasmodial [96], and antifungal properties. Notably, volatile organic compounds (VOCs) from *Nodulisporium* spp. have been reported to inhibit phytopathogens such as *Fusarium oxysporum* in cherry tomatoes [97], *Penicillium* spp. in citrus fruits [98], and others like *Pythium aphanidermatum*, *Rhizoctonia solani*, *Phytophthora cinnamomi*, and *Sclerotinia sclerotiorum* [99,100]. However, VOCs were outside the scope of this study. The low activity observed may be due to the specific conditions tested, which favored non-volatile antifungal mechanisms.

### 3.4. Some Antifungal Compounds from Endophytic Diaporthe sp.

Notably, the endophytic isolates exhibiting the highest antifungal activity against *F. solani* and *F. equiseti* were phylogenetically related with the genus *Diaporthe* (Table 4). This genus, classified within the *phylum* Ascomycota, comprises over 950 described species that function as endophytes, saprophytes, or pathogens in a diverse range of plant and mammalian hosts [8]. Species within this genus are characterized by their ability to produce a broad spectrum of bioactive metabolites, including terpenoids, steroids, macrolides, alkaloids, flavonoids, and polyketides [101]. These metabolites exhibit cytotoxic and antineoplastic effects [102] and anti-inflammatory [103], hypocholesterolemic [104], cytotoxic [105], herbicide [106], antibacterial, and antifungal activities [107].

**Figure 5:**
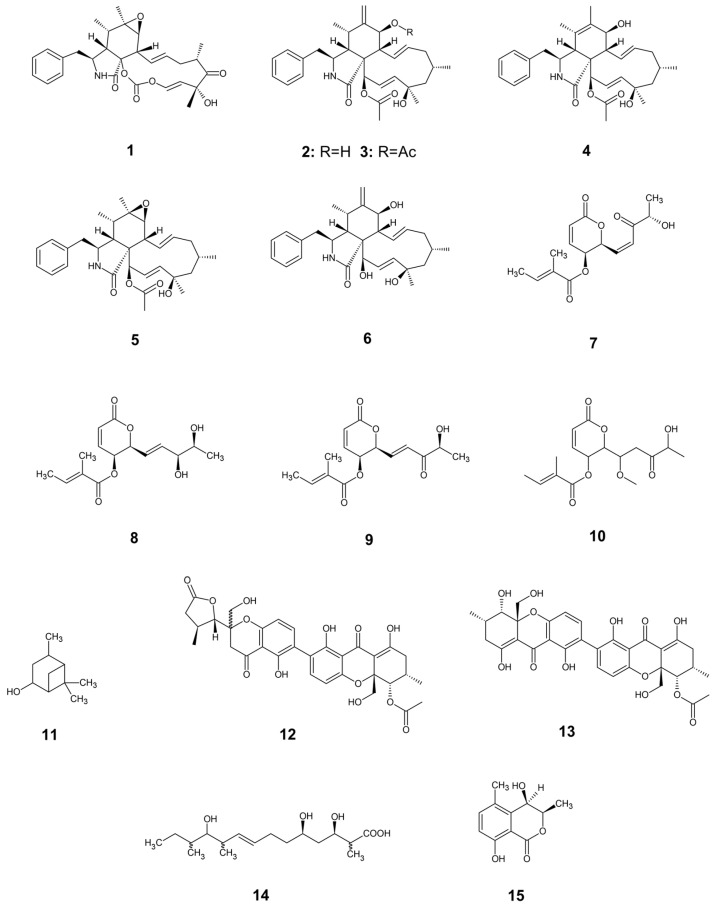
Antifungal metabolites from *Diaporthe* sp. with inhibitory activity on plant phytopathogens.

Among antifungal metabolites, cytochalasin-like compounds are the most abundant in the *Diaporthe* genus (Figure 5). These secondary metabolites, derived from polyketide-amino acid fusion, have demonstrated biocontrol potential. They are known as microfilament-targeting molecules, which exhibit a wide range of biological activities by interfering with several cellular processes involving cytoskeleton formation [108]. For example, Huang et al. reported that cytochalasins E (**1**), H (**2**), and 7-acetoxy-cytochalasin (**3**) from *Diaporthe* sp. exhibit strong antifungal activity against *Alternaria oleracea*, *Pestalotiopsis theae*, and *Colletotrichum capsici* (MIC= 3.125–12.5 μg/mL) [109]. Similarly, cytochalasins H (**2**) and N (**4**) and epoxy-cytochalasin H (**5**) from *Phomopsis* sp. By254 inhibited *Sclerotinia sclerotiorum*, *Fusarium oxysporum*, *Botrytis cinerea*, and *Rhizoctonia cerealis* (IC_50_= 0.1–5.0 μg/mL) [110]. In addition, cytochalasin J (**6**) from *D. miriciae* showed antifungal activity against the fungal plant pathogens *Phomopsis obscurans* and *P. viticola* at 300 µM [111].

*Diaporthe* species produce another important antifungal compound that has been evaluated on plant phytopathogens (Figure 4). Among them, phomompsolide A (**7**), phomopsolide B (**8**), and phomopsolide C (**9**), isolated from *D. maritima* cultures, demostrated growth inhibition on *Microbotryum violaceum* and *Saccharomyces cerevisiae* at 25–250 µM [112]. Likewise, phomopsolide G (**10**) was isolated from *Diaporthe* sp. AC1 and showed moderate antifungal activity against *Fusarium graminearum*, *F. moniliforme*, and *Botrytis cinerea* [113]. Additionally, the monoterpene verbanol (**11**) from *D. terebinthifolii* showed inhibitory activity on the germination of *Phyllosticta citricarpa* conidia [114]. In the same way, two xanthone dimers, diaporxanthone A (**12**) and diaporxanthone B (**13**), produced by *D. goulteri* L17 exhibited moderate antifungal activities against *Nectria* sp. and *Colletotrichum musae* [115]. Gao et al. (2020) isolated from *D. eucalyptorum* a rare polyketide fatty acid, eucalyptacid A (**14**), with MIC values ranging from 12.5 to 50.0 µM against *Alternaria solani*, *B. cinerea*, *F. solani*, and *Gibberella saubinettii* [116]. The dihydroisocoumarin (−)-(3R,4R)-*cis*-4-hydroxy-5-methylmellein (**15**) produced by *D.* cf. *heveae* drastically reduced the growth of *Phyllosticta citricarpa* and *Colletotrichum abscissum*, two important citrus diseases worldwide [117]. Notably, the differences in antifungal activity metrics (MIC, IC_50_) and concentration units (µg/mL vs. µM), derived from the heterogeneity in experimental approaches, limits direct comparison of biological activity data.

### 3.5. Molecular Docking of Diaporthe antifungals with Chitin Synthase

The antifungal activity, characterized by the inhibition of mycelial growth, provided a starting point to investigate the cell wall as the primary target. Consequently, we chose CS as a specific target for cell wall inhibition in the in silico assays. Nevertheless, the following results do not aim to be a confirmatory result of the mechanism of action of these isolates or molecules, but rather a proposal based on molecular evidence.

The amino acid sequences of CS for *F. solani* and *F. equiseti* were retrieved from the UniProt database under accession codes A0A9P9GUZ8 and A0A8J2II09, respectively. A BLASTp search against the PDB identified the cryo-EM structure of *Phytophthora sojae* CS 1 (PDB ID: 7WJO) as the most suitable template for homology modeling, given its high sequence identity and structural coverage. Among the 3D models generated by the different servers, those predicted by Robetta exhibited the highest structural fidelity to the template, displaying the lowest Root Mean Square Deviation (RMSD) values of 1.68 Å for *F. equiseti* and 2.21 Å for *F. solani*. Furthermore, the structural reliability of the models was further validated through various quality metrics (Table S3). While these metrics confirm high geometric consistency, the QMEANDisCo values suggest that future refinements could be possible as more high-resolution templates for fungal membrane enzymes become available. Consequently, these Robetta-derived models were selected as the reference structures for the subsequent molecular docking simulations.

**Figure 6:**
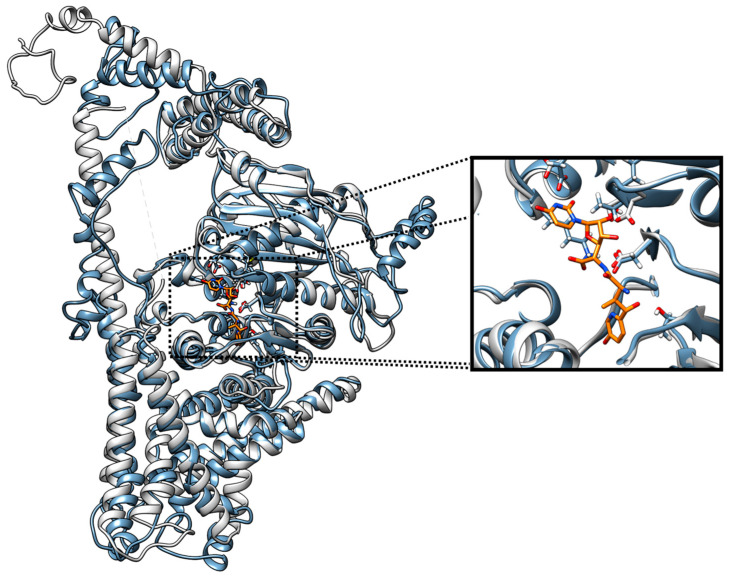
Global superimposition of the generated *F. equiseti* CS model against the *P. sojae* template (PDB: 7WJO). The gray structure displays the CS template (*P. sojae*) and blue displays the *F. equiseti* model. The right side of the picture shows a zoomed-in view of the binding zone.

Visual inspection of the superimposed structures confirms a remarkable topological similarity between the generated *Fusarium* models and the *P. sojae* template. Crucially, the detailed analysis of the active site reveals that the architecture of the ligand-binding pocket is maintained in both *F. solani* and *F. equiseti* models. As depicted in Figure 6, the key amino acid residues responsible for stabilizing the reference inhibitor Nikkomycin Z are spatially conserved. This structural preservation suggests that the generated models possess a binding pocket competent for ligand recognition, thereby validating their use for the subsequent molecular docking of the endophytic metabolites. Additionally, a redocking procedure for Nikkomycin Z yielded an RMSD value of 1.32 Å, confirming that the simulation parameters effectively reproduce the bioactive conformation of the ligand within the active site.

The normalized binding energy values (Vina score) shown in Table 5 revealed that several isolated metabolites possess a theoretical affinity for the CS active site that rivals or even surpasses the reference inhibitor. Notably, compound **12** exhibited the highest predicted binding stability, achieving a normalized Vina score of 1.0 in *F. equiseti* and 0.83 in *F. solani*. These values suggest that compound **12** has the potential to form a complex with the enzyme that is theoretically more favorable than the control Nikkomycin Z (which scored 0.65 and 0.57, respectively). This predictive data implies that the hydrophobic skeleton of the isolated endophyte metabolites likely fits tightly within the catalytic pocket, potentially driven by strong Van der Waals interactions.

While high affinity is crucial, the specific mode of binding determines the biological outcome. The interaction fingerprint analysis (PLIF) quantified how well the candidates mimicked the key contacts established by Nikkomycin Z. The similarity scores for the top candidates ranged between 0.40 and 0.66. While lower than the control (which is 1.0 by definition), these values are significant for non-peptidic small molecules. Specifically, compound **13** showed the highest structural similarity in interaction patterns against *F. equiseti* (0.66), implying that it engages conserved residues essential for catalysis, such as those involved in uridine binding or translocation [118,119]. The lower similarity scores observed for compound **12** (approx. 0.40–0.46), despite its high energy, suggest it may occupy the binding pocket in a unique orientation, potentially exploiting auxiliary sub-pockets not accessed by the reference inhibitor.

**Figure 7:**
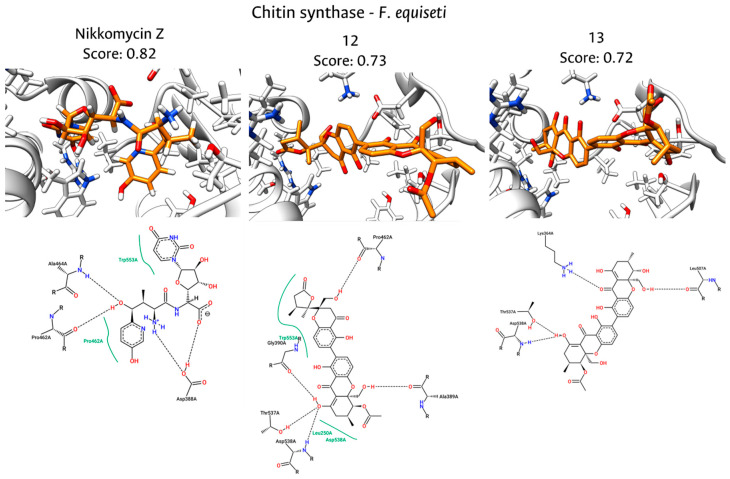
Interactions between the binding site of CS of *F. equiseti* and docked molecules (NikkomycinZ, compounds **12** and **13**). The **upper** part shows the 3D interactions and the **lower** part the 2D interaction plots.

Detailed inspection of the ligand–receptor contacts in *F. equiseti* reveals that Nikkomycin Z (NZ) anchors itself within the active site through hydrogen bonds with Asp388, Pro462, and Ala464, while relying on a critical hydrophobic stacking interaction with Trp553, as is shown in Figure 7. This tryptophan residue is structurally significant, as it corresponds to the conserved aromatic residues found in the catalytic loop of the *P. sojae* template and other Family 2 glycosyltransferases, acting as a gatekeeper that stabilizes the GlcNAc sugar ring [36,120]. Notably, compound **12** successfully mimics this pharmacophoric feature, preserving the hydrophobic clamp with Trp553 and the hydrogen bond with Pro462, while further stabilizing the complex through additional hydrogen bonds with Asp538 and Gly390. The engagement with Asp538 is particularly relevant, as aspartate residues in this region are typically involved in coordinating the divalent metal ions essential for catalysis [121,122].

The structural basis for the consensus ranking becomes evident when comparing these interaction profiles. While compound **13** establishes a strong hydrogen bonding network involving Asp538, Thr537, and Lys364, it notably lacks the hydrophobic interaction with Trp553 observed in both NZ and compound **12**. This absence likely accounts for its slightly lower thermodynamic stability (Vina score) compared to compound **12**, despite having a high interaction similarity index. Consequently, compound **12** could have better activity (consensus score = 0.73) because it acts as a dual-modality inhibitor: it not only occupies the catalytic center by interacting with essential aspartates but also replicates the hydrophobic stabilization mechanism exploited by the reference inhibitor.

**Figure 8:**
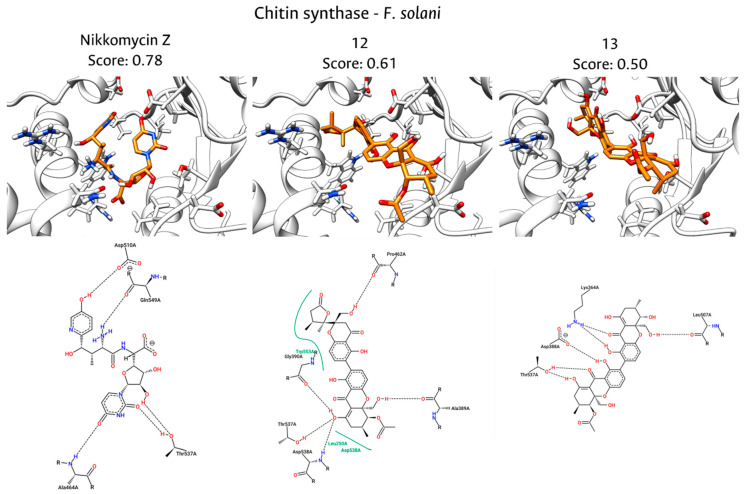
Interactions between the binding site of CS of *F. solani* and docked molecules (NikkomycinZ, compounds **12** and **13**). The **upper** part shows the 3D interactions and the **lower** part the 2D interaction plots.

The docking results for *F. solani* reveal a distinct interaction landscape, likely attributed to the greater structural divergence of its catalytic domain relative to the template. This conformational variability is reflected in the binding mode of Nikkomycin Z, which shifts to a pose dominated by hydrogen bonds with Asp510, Gln549, Thr537, and Ala464, notably losing the strong hydrophobic anchor observed in the *F. equiseti* model, as is displayed in Figure 8. In contrast, compound **12** demonstrates remarkable adaptability to this distorted pocket. It not only establishes a wide hydrogen bonding network with Pro462, Thr537, Asp538, Ala389, and Gly690, but crucially, it recovers the hydrophobic stacking interaction with Trp553. This ability explains the predicted binding energy (0.83 and 0.57 for compound **12** and Nikkomycin, respectively).

Compound **13** relies primarily on electrostatic and polar stabilization, anchoring to the active site through hydrogen bonds with Lys364, Asp388, and Thr537. While Thr537 emerges as persistent contact across all ligands, suggesting a pivotal role in substrate orientation, the absence of the hydrophobic clamp with Trp553 limits the binding stability of compound **13**. This comparison further validates the consensus scoring hierarchy, identifying compound **12** as the most robust candidate capable of maintaining key inhibitory interactions despite the structural plasticity of the pathogen’s target site for CS in both organisms. The strong correlation between the high consensus docking scores and the arrested hyphal growth suggests that these compounds exert their antifungal effect by targeting the chitin biosynthetic machinery, thereby compromising the structural integrity of the fungal cell wall. Nevertheless, more studies are needed to verify this theory.

### 3.6. Concluding Remarks

Antillean avocado trees from Montes de María, which have survived under adverse phytosanitary conditions, host a diverse community of endophytic fungi, some of which produce antifungal metabolites active against *F. solani* and *F. equiseti*. The most active isolates, identified as *Diaporthe* spp., were found primarily in Manteca and Cebo ecotypes, suggesting a possible correlation between endophyte presence and host resilience. The taxonomic framework provided by ITS allowed for the successful identification of the *Diaporthe* genus; however, further genetic characterization is necessary to confirm the precise species of these promising bioactive strains. These findings underscore the potential of *Diaporthe* endophytes as a natural source of bioactive compounds for plant disease management. While in vitro results are promising, they must be complemented with in planta validation, such as greenhouse-based protection assays on avocado seedlings, to assess the efficacy of the extracts or isolates under realistic physiological conditions. At the same time, bioassay-guided fractionation and chemical characterization are critical to identify the specific antifungal metabolites and evaluate their potential for practical applications.

The in silico analysis of CS highlighted the potential inhibitory activity of two diaporxanthones, which showed strong predicted binding affinity and may contribute to the antifungal effects observed in vitro. Although these models offer valuable theoretical insight, their conclusions rely on homology-based structures and should be interpreted with caution. To strengthen this approach, subsequent investigations must prioritize direct enzymatic inhibition assays to experimentally confirm the proposed affinity and mechanisms, alongside structural biology efforts aimed at crystallizing the specific *Fusarium* CS to validate predicted binding modes. Together, the integration of ecological, in vitro, and computational findings provides a strong foundation for exploring the biotechnological potential of endophytic fungi from resilient avocado ecotypes, while clearly outlining the next steps toward experimental and applied validation.

## Figures and Tables

**Table 1:** Molecular characterization and GenBank accession numbers for isolates from secondary roots of Antillean avocado trees with disease symptoms.

Fungal Isolate	Accession Number	Closest Related Species	Similarity (%)
UN02	PP052962	OQ673588.1 *Epicoccum* sp.	100.00
UN23	PP052963	MG980304.1 *Colletotrichum* sp.	100.00
UN29	OQ271226.1	MN989030.1 *Fusarium solani*	100.00
UN31	PP052964	NR_130690.1 *Fusarium* sp.	100.00
UN36	PP052965	LC406903.1 *Colletotrichum* sp.	99.65
UN37	OQ344629.1	OP006756.1 *Fusarium equiseti*	100.00

**Table 2:** Mycelial growth inhibition (%MGI) of endophytic extract at 1000 µg/mL against *Fusarium* pathogens. Data shows media value ± standard deviation. N/E: Not evaluated (endophytes did not show growth inhibition zones of pathogens in dual cultures).

Endophytic EtOAc Extract N.	% MGI		Endophytic EtOAc Extract N.	% MGI	
	*F. solani*	*F. equiseti*		*F. solani*	*F. equiseti*
**UN22**	**66.2 ± 0.5 ^h,A^**	**68.6 ± 2.0 ^a,A^**	UN100	12.8 ± 2.5 ^e^	27.1 ± 0.6 ^h^
**UN39**	**57.7 ± 1.2 ^i^**	**70.9 ± 3.2 ^a^**	UN102	7.3 ± 1.1 ^a,d^	15.5 ± 2.1 ^j^
**UN51**	50.2 ± 0.3	**57.1 ± 3.3 ^b^**	UN103	6.2 ± 0.9 ^a,d^	36.7 ± 1.6 ^f^
UN68	5.6 ± 2.9 ^a^	30.3 ± 2.1 ^g^	UN104	−6.8 ± 1.0 ^c^	N/E
UN70	0.9 ± 2.2 ^b,B^	3.0 ± 1.7 ^B^	UN105	35.1 ± 1.0	39.1 ± 2.5 ^d,e^
UN71	3.8 ± 2.5 ^a,b^	8.9 ± 1.5 ^k^	UN106	−3.6 ± 1.0 ^c^	17.7 ± 1.1 ^i,j^
UN76	−5.1 ± 1.4 ^c^	21.3 ± 3.4 ^i^	UN107	24.9 ± 1.3 ^g^	43.8 ± 2.5 ^e^
UN77	41.2 ± 1.2 ^k^	46.2 ± 2.0 ^d^	UN112	11.5 ± 2.3 ^e^	18.7 ± 0.7 ^i,j^
UN79	2.4 ± 2.7 ^a,b^	13.5 ± 3.1 ^j^	UN113	13.2 ± 1.2 ^e^	24.1 ± 1.2 ^h^
UN80	18.9 ± 3.2 ^f,C^	20.8 ± 2.7 ^i,C^	UN115	8.0 ± 2.2 ^a,d,e^	21.7 ± 2.6 ^i^
UN82	24.5 ± 3.1 ^g^	N/E	UN122	25.6 ± 1.8 ^g^	29.0 ± 1.6 ^g,h^
UN86	4.3 ± 2.4 ^a,D^	6.7 ± 2.2 ^k,D^	UN123	14.0 ± 2.2 ^e^	25.3 ± 1.7 ^h^
UN87	18.3 ± 1.8 ^f^	25.9 ± 3.1 ^h^	UN301	9.7 ± 1.3 ^d,e^	21.0 ± 0.8 ^i^
**UN92**	**70.1 ± 1.6 ^j^**	**57.1 ± 2.0 ^b^**	UN302	22.8 ± 0.7 ^g^	28.0 ± 2.7 ^g^
**UN93**	**63.1 ± 0.7 ^h^**	19.9 ± 1.67	**UN310**	**60.8 ± 1.6 ^h,i,E^**	**61.3 ± 1.0 ^b,c,E^**
UN94	7.4 ± 1.5 ^a,d^	N/E	UN314	14.3 ± 1.6 ^e,f,F^	16.0 ± 2.6 ^j,F^
**UN95**	**60.7 ± 0.9 ^h,i^**	51.1 ± 3.7 ^e^	UN318	18.0 ± 1.6 ^f^	21.6 ± 1.8 ^i^
**UN99**	**59.6 ± 2.9 ^h,i^**	**53.4 ± 0.5 ^e^**	---	---	---

Values followed by the same lowercase letters in the columns of % MGI for each pathogen at 1000 µg/mL do not show significant differences according to Tukey’s test (*p* > 0.05). Similarly, means followed by the same uppercase letters in the rows are not significantly different in a one-way ANOVA analysis (*p* > 0.05). Bold: most promising antifungal extracts.

**Table 3:** Dose–response curves and median inhibitory concentration (IC_50_) values of EtOAc extracts which showed % MGI > 50% on *Fusarium* phytopathogens.

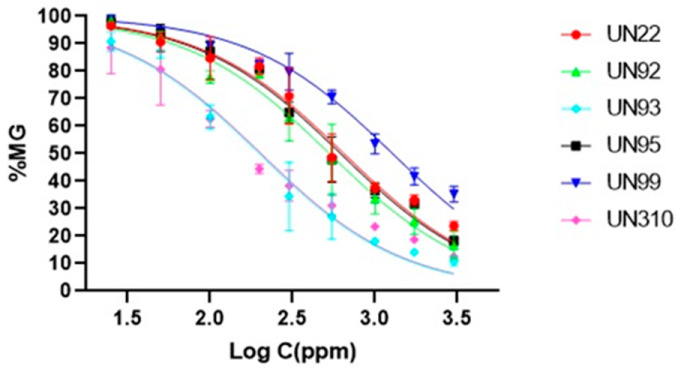		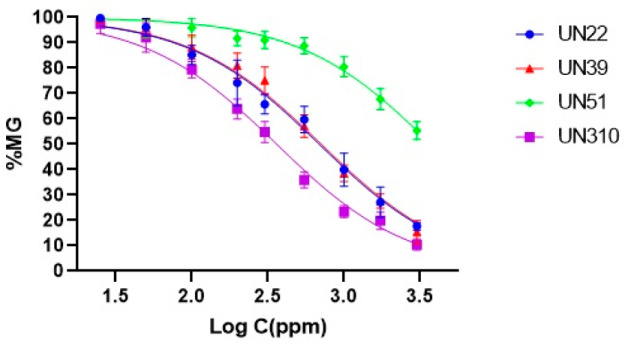	
**Endophytic** **EtOAc N.**	***F. solani***		***F. equiseti***
	**IC_50_ (µg/mL)**		**IC_50_ (µg/mL)**
UN22	650 ± 69 ^b,B^		688 ± 50 ^a,B^
UN39	N/D		710 ± 43 ^a^
UN51	N/D		˃3000
UN92	531 ± 51 ^b^		N/D
UN93	**199 ± 29 ^c^**		N/D
UN95	550 ± 40 ^b^		N/D
UN99	1281 ± 78 ^a^		N/D
UN310	**204 ± 25 ^c,D^**		362 ± 27 ^b,D^

Values followed by the same lowercase letters in the columns of % MGI for each pathogen at 1000 µg/mL do not show significant differences according to Tukey’s test (*p* > 0.05). Similarly, means followed by the same uppercase letters in the rows are not significantly different in a one-way ANOVA analysis (*p* > 0.05). N/D: Not determined.

**Table 4:** Identification of active endophytic fungi isolated from Antillean avocado leaves.

Fungal Isolate	Accession Number	Closest Related Species	Similarity (%)
UN22	OQ914368	MW566594.1 *Diaporthe ueckeri*	99
UN39	OQ914369	MW380843.1 *Diaporthe phaseolorum*	100
UN51	OQ914370	EF694672.1 *Nodulisporium* sp.	96
UN92	OQ914371	MW380843.1 *Diaporthe phaseolorum*	100
UN93	OQ914372	KF498865.1 *Diaporthe phaseolorum*	100
UN95	OQ914373	KX631735.1 *Diaporthe longicolla*	100
UN99	OQ914374	AY577815.1 *Diaporthe phaseolorum*	100
UN310	OQ914375	KF498865.1 *Diaporthe phaseolorum*	99

**Table 5:** Scoring table of molecular docking simulations of each metabolite against the CS *F. solani* and *F. equiseti*. In the consensus score column, the green color displays the best scores, the red color the worst scores, and yellow colors intermediate scores.

	*F. solani*			*F. equiseti*		
id	Vina Score	PROLIF Similarity	Consensus Score	Vina Score	PROLIF Similarity	Consensus Score
Nikkomycin Z	0.57	1.00	0.78	0.65	1.00	0.82
Cytochalasin E (**1**)	0.27	0.63	0.45	0.37	0.48	0.42
Cytochalasin H (**2**)	0.16	0.49	0.32	0.30	0.38	0.34
7-acetoxy-cytochalasin (**3**)	0.22	0.75	0.49	0.26	0.85	0.56
Cytochalasin N (**4**)	0.24	0.44	0.34	0.38	0.75	0.57
Epoxy-cytochalasin H (**5**)	0.26	0.67	0.46	0.29	0.57	0.43
Cytochalasin J (**6**)	0.23	0.69	0.46	0.38	0.59	0.48
Phomompsolide A (**7**)	0.31	0.42	0.37	0.37	0.55	0.46
Phomompsolide B (**8**)	0.40	0.56	0.48	0.24	0.55	0.39
Phomompsolide C (**9**)	0.34	0.51	0.43	0.25	0.49	0.37
Phomompsolide G (**10**)	0.16	0.19	0.17	0.33	0.45	0.39
Verbanol (**11**)	0.00	0.28	0.14	0.00	0.51	0.26
Diaporxanthone A (**12**)	1.00	0.22	0.61	0.96	0.50	0.73
Diaporxanthone B (**13**)	0.71	0.29	0.50	1.00	0.45	0.72
Eucalyptacid A (**14**)	0.04	0.00	0.02	0.41	0.00	0.20
Dihydroisocoumarin (−)-(3R,4R)-cis-4-hydroxy-5-methylmellein (**15**)	0.18	0.14	0.16	0.18	0.46	0.32

## Data Availability

The authors declare that the data supporting the findings of this study are available within the paper and its Supplementary Information Files. Should any raw data files be needed in another format, they are available from the corresponding author upon reasonable request. All ITS-rDNA sequences were submitted to GenBank to obtain accession numbers (Table S1).

## Supplementary Materials

The following supporting information can be downloaded at: https://www.mdpi.com/article/10.3390/jof12010052/s1. Table S1: DNA sequences for ITS1 region of *Fusarium* pathogens and promising endophytes; Table S2: Extraction yields for antifungal endophytes fermentation using EtOAc; Table S3: Metrics for validation of homology models. Reference [57] is cited in the Supplementary Materials.

## Abbreviations

The following abbreviations are used in this manuscript:

AWC	Avocado Wilt Complex
CS	Chitin Synthase
DMSO	Dimethyl Sulfoxide
EtOAc	Ethyl Acetate
IC_50_	Median Inhibitory Concentration
ITS	Internal Transcribed Spacer
MGI	Mycelial Growth Inhibition
MIC	Minimal Inhibitory Concentration
NZ	Nikkomycin Z
PDA	Potato Dextrose Agar
