# Supplementary material for: Antifungal Potential of Diaporthe sp. Endophytes from Antillean Avocado Against Fusarium spp.: From Organic Extracts to In Silico Chitin Synthase Inhibition
Source: J Fungi (Basel). 2026 Jan 11;12(1):52. doi: 10.3390/jof12010052 (PMC12843151; doi:10.3390/jof12010052)
Supplement: Supplementary file 1 [file jof-12-00052-s001.zip › jof-4057562-supplementary.pdf]

Antifungal Potential of *Diaporthe* sp. Endophytes from Antillean Avocado Against *Fusarium* spp.: From Organic Extracts to In Silico Chitin Synthase Inhibition

Angie T. Robayo-Medina [1,2,*], Katheryn Michell Camargo-Jimenez [1], Felipe Victoria-Muñoz [2], Wilman Delgado-Avila [1], Luis Enrique Cuca [1] and Mónica Ávila-Murillo [1,*]

[1] Grupo de Investigación Estudio Químico de Productos Naturales Vegetales Bioactivos—QUIPRONAB, Departamento de Química, Facultad de Ciencias, Universidad Nacional de Colombia, Sede Bogotá, Bogotá D.C. 111321, Colombia; kmcamargoji@unal.edu.co (K.M.C.-J.); wadelgadoa@unal.edu.co (W.D.-A.); lecucas@unal.edu.co (L.E.C.)
[2] Grupo de Investigación FarmaBioTech, Facultad de Ciencias Exactas y Naturales, Fundación Universitaria Salesiana, Bogotá D.C. 111071, Colombia; daniel.victoria@salesiana.edu.co (F.V.-M.)
* Correspondence: angie.robayo@salesiana.edu.co (A.T.R.-M.); mcavilam@unal.edu.co (M.Á.-M.)

Supplementary material

Page

Table S1. DNA sequences for ITS1 region of *Fusarium* pathogens and antifungal endophytes **S2**
Table S2. Extraction yields for antifungal endophytes fermentation using EtOAc **S4**
Table S3. Metrics for validation of homology models S5

References **S5**

Table S1. DNA sequences for ITS1 region of *Fusarium* pathogens and antifungal endophytes.

<table>
<tr><th>Isolated strain</th><th>GenBank Accession</th><th>Sequence identification [1]</th></tr>
<tr><td>UN29</td><td>OQ271226.1</td><td>Fusarium solani (pathogen)</td></tr>
<tr><td colspan="3">TCACCCTGTGACATACCTATAACGTTGCCTCGGCGGGAACAGACGGCCCCGTAACACGGGCCGCCCCCGCCAGA
GGACCCCCTAACTCTGTTTCTATAATGTTTCTTCTGAGTAAACAAGCAAATAAATTAAAACTTTCAACAACGGATC
TCTTGGCTCTGGCATCGATGAAGAACGCAGCGAAATGCGATAAGTAATGTGAATTGCAGAATTCAGTGAATCATC
GAATCTTTGAACGCACATTGCGCCCGCCAGTATTCTGGCGGGCATGCCTGTTCGAGCGTCATTACAACCCTCAGG
CCCCCGGGCCTGGCGTTGGGGATCGGCGGAAGCCCCCTGCGGGCACAACGCCGTCCCCCAAATACAGTGGCGGT
CCCGCCGCAGCTTCCATTGCGTAGTAGCTAACACCTCGCAACTGGAGAGCGGCGCGGCCACGCCGTAAAACACC
CAACTTCTGAATGTTGACCTCGAATCAGGTAGGAATACCCGCTGAACTTAAGCATATCAATAAGCGGAGGAA</td></tr>
<tr><td>UN37</td><td>OQ344629.1</td><td>Fusarium equiseti (pathogen)</td></tr>
<tr><td colspan="3">CGGGACGGCCCGCCCGAGGACCCTAAACTCTGTTTTTAGTGGAACTTCTGAGTAAAACAAACAAATAAATCAAA
ACTTTCAACAACGGATCTCTTGGTTCTGGCATCGATGAAGAACGCAGCAAAATGCGATAAGTAATGTGAATTGCA
GAATTCAGTGAATCATCGAATCTTTGAACGCACATTGCGCCCGCCAGTATTCTGGCGGGCATGCCTGTTCGAGCGT
CATTTCAACCCTCAAGCTCAGCTTGGTGTTGGGACTCGCGGTAACCCGCGTTCCCCAAATCGATTGGCGGTCACGT
CGAGCTTCCATAGCGTAGTAATCATACACCTCGNTACTGTATCGTCGCGGCCACGCCGT</td></tr>
<tr><td>UN22</td><td>OQ914368</td><td>Diaporthe ueckeri (endophyte)</td></tr>
<tr><td colspan="3">TGGGGGTTTAACGGCAGGGCACCGCCAGGGCCTTCCAGAACGAGATATAACTACTACGCTCGGGGTCCTAGCGA
GCTCGCCACTAGATTTCAGGGCCTGCCCTCGCTAGAAGGCAGTGCCCCATCACCAAGCCAGGCTTGAGGGTTGAA
ATGACGCTCGAACAGGCATGCCCTCCGGAATACCAGAGGGCGCAATGTGCGTTCAAAGATTCGATGATTCACTG
AATTCTGCAATTCACATTACTTATCGCATTTCGCTGCGTTCTTCATCGATGCCAGAACCAAGAGATCCGTTGTTGA
AAGTTTTGATTCATTTATGTTTTTTACTCAGAGATTCACTATAGAAACAAGAGTTTAGTTGGCCGCCGGCGGGCTG
CTCCCTGTTTCCAGGGGGCCTCAGTGAAGAGGCCGGCCTACGCCGAGGCAACAAATAGGTATAAGTTCACAAAG
GGTTTCTGGGTGCGCCGAAGCGCGTTCCAGCAATGATCCCTCCGCTGGTTCACCAACGGAGACCTTGTTACGACTT
TTTACTTC</td></tr>
<tr><td>UN39</td><td>OQ914369</td><td>Diaporthe phaseolorum (endophyte)</td></tr>
<tr><td colspan="3">AATTTTCAGAAGTTGGGGGTTTAACGGCAGGGCACCGCCAGGGCCTTCCAGAACGAGATATAACTACTACGCTCG
GGGTCCTAGCGAGCTCGCCACTAGATTTCAGGGCCTGCCCTCGTTAGAAGGCAGTGCCCCATCACCAAGCCAGGC
TTGAGGGTTGAAATGACGCTCGAACAGGCATGCCCTCCGGAATACCAGAGGGCGCAATGTGCGTTCAAAGATTC
GATGATTCACTGAATTCTGCAATTCACATTACTTATCGCATTTCGCTGCGTTCTTCATCGATGCCAGAACCAAGAG
ATCCGTTGTTGAAAGTTTTGATTCATTTATGTTTTTACTCAGAGATTCACTATAGAAACAAGAGTTTAGTTGGCCGC
CGGCGGGCTGCTCCCTGTTTCCAGGGGGCCTCAGTGAAGAGGCCGGCCTACGCCGAGGCAACAAATAGGTATAA
GTTCACAAAGGGTTTCTGGGTGCGCCGAAGCGCGTTCCAGCAATGATCCCTCCGCTGGTTCACCAACGGAGACCT
TGTTACGACTTTTTACTT</td></tr>
<tr><td>UN51</td><td>OQ914370</td><td>Nodulisporium sp. (endophyte)</td></tr>
<tr><td colspan="3">ATAGGGGGTTTTACGGCCAGCAGCCAGGGCCACCACACGAGCGAGAGAGATTACTACGCTGAGAGTGTACCCTA
ACTCCGCCACTGATTTTCAGGAACTACGCCGAAGCCGTAGACTCCCAACACTAAGCAACAGGGCTTAAGGGTTG
AAATGACGCTCGAATAGGCATGCCCACTAGAATACTAGTGGGCGCAATGTGCGTTCAAAGATTCGATGATTCACT
GAATTCTGCAATTCACATTACTTATCGCATTTCGCTGCGTTCTTCATCGATGCCAGAACCAAGAGATCCGTTGTTG
AAAGTTTTAACTTATTTCAGTTACGTGTTCAGAGATACAGTTGTAGAAGCAAGAGTTTAACGGTCCTTCGGCGGGC
CTTCACGCGGCTACAGGGTAGCTCCAGGGTAGACAACTACTACAGGGTAGGTGCCTGCCAAGATAACTCCTGGC
AGACAACCACTACAGAGGTAGGTGCCTGACAGGATAGCTCTTGGCAGACAACCACTACAGGGTAGGTGCCTTCC
AGGGTAGCTCCAGGTAGCAGCAGCACGCGCCGAGGAAACGACGGTAAGGTTCACAAAGGGTTTTGGAGTTTAGT
AACTCATTAATGATCCCTCCGCTGGTTCACCAACGGAGACCTTGTTACGACTTTTACTTC</td></tr>
<tr><td>UN92</td><td>OQ914371</td><td>Diaporthe phaseolorum (endophyte)</td></tr>
<tr><td colspan="3">GAAGTTGGGGGTTTAACGGCAGGGCACCGCCAGGGCCTTCCAGAACGAGATATAACTACTACGCTCGGGGTCCT
AGCGAGCTCGCCACTAGATTTCAGGGCCTGCCCTCGTTAGAAGGCAGTGCCCCATCACCAAGCCAGGCTTGAGG
GTTGAAATGACGCTCGAACAGGCATGCCCTCCGGAATACCAGAGGGCGCAATGTGCGTTCAAAGATTCGATGAT
TCACTGAATTCTGCAATTCACATTACTTATCGCATTTCGCTGCGTTCTTCATCGATGCCAGAACCAAGAGATCCGTT
GTTGAAAGTTTTGATTCATTTATGTTTTTACTCAGAGATTCACTATAGAAACAAGAGTTTAGTTGGCCGCCGGCGG
GCTGCTCCCTGTTTCCAGGGGGCCTCAGTGAAGAGGCCGGCCTACGCCGAGGCAACAAATAGGTATAAGTTCAC
AAAGGGTTTCTGGGTGCGCCGAAGCGCGTTCCAGCAATGATCCCTCCGCTGGTTCACCAACGGAGACCTTGTTAC
GACTTTTTACTTCC</td></tr>
</table>

UN93	OQ914372	*Diaporthe phaseolorum* (endophyte)
GAAGTTGGGGGTTTAACGGCAGGGCACCGCCAGGGCCTTCCAGAACGAGATATAACTACTACGCTCGGGGTCCT AGCGAGCTCGCCACTAGATTTCAGGGCCTGCCCTCGCTAGAAGGCAGTGCCCCATCACCAAGCCAGGCTTGAGG GTTGAAATGACGCTCGAACAGGCATGCCCTCCGGAATACCAGAGGGCGCAATGTGCGTTCAAAGATTCGATGAT TCACTGAATTCTGCAATTCACATTACTTATCGCATTTCGCTGCGTTCTTCATCGATGCCAGAACCAAGAGATCCGTT GTTGAAAGTTTTGATTCATTTATGTTTTTACTCAGAGATTCACTATAGAAACAAGAGTTTAGTTGGCCGCCGGCGG GCTGCTCCCTGTCTCCAGGGGGCCTCAGTGAAGAGGCCGGCCTGCGCCGAGGCAACAGATAGGTATAAGTTCAC AAAGGGTTTCTGGGTGCGCCGAAGCGCGTTCCAGCAATGATCCCTCCGCTGGTTCACCAACGGAGACCTTGTTAC GACTTTTACT		
UN95	OQ914373	*Diaporthe longicolla* (endophyte)
GGGGGTTTAACGGCAGGGCACCGCCAGGGCCTTCCAGAACGAGATATAACTACTACGCTCGGGGTCCTAGCGAG CTCGCCACTAGATTTCAGGGCCTGCCCTCGCTAGAAGGCAGTGCCCCATCACCAAGCCAGGCTTGAGGGTTGAAA TGACGCTCGAACAGGCATGCCCTCCGGAATACCAGAGGGCGCAATGTGCGTTCAAAGATTCGATGATTCACTGA ATTCTGCAATTCACATTACTTATCGCATTTCGCTGCGTTCTTCATCGATGCCAGAACCAAGAGATCCGTTGTTGAA AGTTTTGATTCATTTATGTTTTTACTCAGAGATTCACTATAGAAACAAGAGTTTAGTTGGCCGCCGGCGGGCTGCT CCCTGTCTCCAGGGGGCCTTTGTCACAAAAGGCCGGCCTGCGCCGAGGCAACAGATAGGTATAAGTTCACAAAG GGTTTCTGGGTGCGCCGAAGCGCGTTCCAGCAATGATCCCTCCGCTGGTTCACCAACGGAGACCTTGTT		
UN99	OQ914374	*Diaporthe phaseolorum* (endophyte)
GAAGTTGGGGGTTTAACGGCAGGGCACCGCCAGGGCCTTCCAGAACGAGATATAACTACTACGCTCGGGGTCCT AGCGAGCTCGCCACTAGATTTCAGGGCCTGCCCCCGTTAGAAGGCAGTGCCCCATCACCAAGCCAGGCTTGAGG GTTGAAATGACGCTCGAACAGGCATGCCCTCCGGAATACCAGAGGGCGCAATGTGCGTTCAAAGATTCGATGAT TCACTGAATTCTGCAATTCACATTACTTATCGCATTTCGCTGCGTTCTTCATCGATGCCAGAACCAAGAGATCCGTT GTTGAAAGTTTTGATTCATTTATGTTTTTACTCAGAGATTCACTATAGAAACAAGAGTTTAGTTGGCCGCCGGCGG GCTGCTCCCTGTCTCCAGGGGGCCTCAGTGAAGAGGCCGGCCTACGCCGAGGCAACAAATAGGTATAAGTTCAC AAAGGGTTTCTGGGTGCGCCGAAGCGCGTTCCAGCAATGATCCCTCCGCTGGTTCACCAACGGAGACCTTGTTAC GACTTTTACTTC		
UN310	OQ914375	*Diaporthe phaseolorum* (endophyte)
GACTACTGATCGAGGTCAATTTTCAGAAGTTGGGGGTTTAACGGCAGGGCACCGCCAGGGCCTTCCAGAACGAG ATATAACTACTACGCTCGGGGTCCTAGCGAGCTCGCCACTAGATTTCAGGGCCTGCCCTCGCTAGAAGGCAGTGC CCCATCACCAAGCCAGGCTTGAGGGTTGAAATGACGCTCGAACAGGCATGCCCTCCGGAATACCAGAGGGCGCA ATGTGCGTTCAAAGATTCGATGATTCACTGAATTCTGCAATTCACATTACTTATCGCATTTCGCTGCGTTCTTCATC GATGCCAGAACCAAGAGATCCGTTGTTGAAAGTTTTGATTCATTTATGTTTTTACTCAGAGATTCACTATAGAAA CAAGAGTTTAGTTGGCCGCCGGCGGGCTGCTCCCTGTCTCCAGGGGGCCTCAGTGAAGAGGCCGGCCTACGCCGA GGCAACAAATAGGTATAAGTTCACAAAGGGTTTCTGGGTGCGCCGAAGCGCGTTCCAGCAATGATCCCTCCGCTG GTTCACCAACGGAGACCTTGTTACGACTTTTTACTTCCA		

Table S2. Extraction yields for antifungal endophytes fermentation using EtOAc. Dry biomass is expressed as the average of dry biomass produced by the endophyte in four flasks with 100 mL of yeast extract broth 2%, at 28°C for 14 days.

Endophyte N.	Dynamic Fermentation		Static Fermentation	
	Dry biomass (g)	Extract/100 mL YEB 2% (mg)	Dry biomass (g)	Extract/100 mL YEB 2% (mg)
UN22	0,522 ± 0,022	17,9	0,499 ± 0,045	23,6
UN39	0,654 ± 0,050	15,6	0,724 ± 0,050	31,2
UN51	0,477 ± 0,116	30,5	0,244 ± 0,087	25,6
UN92	0,824 ± 0,060	27,9	0,627 ± 0,074	35,9
UN93	0,485 ± 0,058	44,7	0,402 ± 0,082	29,3
UN95	0,566 ± 0,045	45,3	0,498 ± 0,027	62,2
UN99	0,520 ± 0,050	107,0	0,334 ± 0,72	77,7
UN310	0,696 ± 0,066	73,5	0,594 ± 0,042	75,5

Table S3. Quality metrics for homology models of Chitin Synthase of *F. solani* and *F. equiseti*.

Metric	*F. solani*	*F. equiseti*
Percentage identity	38.3	42.7
RMSD	2.21	1.68
MolProbity score	1.64	1.43
Ramachandran Favored	91.55	95.15
Ramachandran Outliers	2.05	1.76
QMEANDisCo Global	0.32	0.40

References

[1] A. Morgulis, G. Coulouris, Y. Raytselis, T. L. Madden, R. Agarwala, and A. A. Schäffer, "Database indexing for production MegaBLAST searches," *Bioinformatics*, vol. 24, no. 16, pp. 1757–1764, Aug. 2008, doi: 10.1093/bioinformatics/btn322.
